# Phase III Study of ^18^F-PSMA-1007 Versus ^18^F-Fluorocholine PET/CT for Localization of Prostate Cancer Biochemical Recurrence: A Prospective, Randomized, Crossover Multicenter Study

**DOI:** 10.2967/jnumed.122.264743

**Published:** 2023-04

**Authors:** Pierre Olivier, Anne-Laure Giraudet, Andrea Skanjeti, Charles Merlin, Pierre Weinmann, Ines Rudolph, Alexander Hoepping, Mathieu Gauthé

**Affiliations:** 1 Nuclear Medicine and Nancyclotep Molecular Imaging Platform, CHRU-Nancy, Université de Lorraine, Nancy, France;; 2 Nuclear Medicine, LUMEN, Centre Leon Berard, Lyon, France;; 3 Nuclear Medicine, HCL, Claude Bernard University-Lyon-1, Lyon, France;; 4 Nuclear Medicine, Centre Hospitalier Sud Francilien, Corbeil Essonne, France;; 5 Department of Nuclear Medicine, Centre Jean Perrin, Clermont-Ferrand, France;; 6 HEGP-AP-HP, Nuclear Medicine, Université de Paris, Paris, France;; 7 ABX Advanced Biochemical Compounds, Radeberg, Germany; and; 8 Hôpital Tenon-AP-HP, Sorbonne Université, Paris, France

**Keywords:** PET/CT, prostatic neoplasms, prostate-specific antigen, decision making

## Abstract

The objective of this study was to compare ^18^F-PSMA-1007 PET/CT and ^18^F-fluorocholine PET/CT for the localization of prostate cancer (PCa) biochemical recurrence. **Methods:** This prospective, open-label, randomized, crossover multicenter study included PCa patients with prior definitive therapy and suspected PCa recurrence. All men underwent both ^18^F-PSMA-1007 PET/CT and ^18^F-fluorocholine PET/CT (102 received ^18^F-PSMA-1007 PET/CT first and 88 received ^18^F-fluorocholine PET/CT first). All images were assessed independently by 3 readers masked to all clinical information using a 3-point qualitative scale (0 = no recurrence, 1 = undetermined, and 2 = recurrence). Patients were monitored for approximately 6 mo. An independent panel with a urologist, radiologist, and nuclear physician reviewed all clinical data, including imaging and response to therapy, but were masked regarding PET/CT information; acting in consensus, they determined a patient-based and region-based composite standard of truth for PCa lesions. The “correct detection rates” for PCa lesions on a patient basis for each radiopharmaceutical were compared for the 3 readers individually and for the “average reader.” Secondary objectives included determining whether PET/CT findings affected diagnostic thinking (impact of a test result on posttest vs. pretest probability of a correct diagnosis), therapeutic decision making (description and quantification of impact of diagnostic information gained with both radiopharmaceuticals on patient management), and adequacy of management changes. **Results:** A total of 190 patients were included. The primary endpoint was met. The overall correct detection rates were 0.82 for ^18^F-PSMA-1007 and 0.65 for ^18^F-fluorocholine (*P* < 0.0001) when undetermined findings were considered positive for malignancy and 0.77 and 0.57, respectively (*P* < 0.0001), when undetermined findings were considered negative for malignancy. A change in diagnostic thinking due to PET/CT was reported in 149 patients; ^18^F-PSMA-1007 contributed more than ^18^F-fluorocholine in 93 of these patients. In 122 patients, PET/CT led to an adequate diagnosis that benefited the patient; ^18^F-PSMA-1007 contributed more than ^18^F-fluorocholine in 88 of these patients. **Conclusion:** ^18^F-PSMA-1007 PET/CT is superior to ^18^F-fluorocholine PET/CT for the localization of PCa recurrence. Decision making was more beneficial when based on ^18^F-PSMA-1007 PET/CT results.

## Visual Abstract

**Figure:**
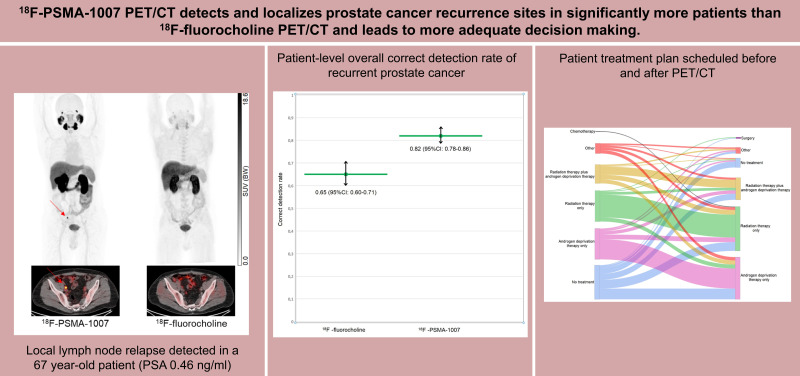

Prostate cancer (PCa) is the most prevalent cancer in men, with approximately 473,000 new diagnoses and more than 108,000 deaths in Europe in 2020 (1). Although long-term outcomes are good for most men, recurrence after definitive therapy is common. One study found that 37% of patients with radical prostatectomy and 48% of patients with radiation therapy had biochemical recurrence within 15 y of the initial definitive treatment; for both, most relapses occurred within the first 5 y (2). The diagnosis of PCa recurrence after prior definitive therapy is based on an increase in serum prostate-specific antigen (PSA); the threshold level varies by treatment, being higher for patients treated with radiation than for those treated by radical prostatectomy (3). PET/CT imaging is the recommended modality for the localization of PCa recurrence (3). ^18^F-fluorocholine has been recommended for PET/CT imaging of PCa recurrence since a marketing authorization was granted in France in 2010. The development of radiopharmaceuticals that directly target the extracellular domain of the prostate-specific membrane antigen (PSMA) resulted in an improvement in the detection of PCa lesions, and this approach is now recommended for PET/CT imaging of PCa recurrence.

A recent metaanalysis evaluated the diagnostic efficacy of all PSMA-directed PET agents and reported an overall detection rate of 74.1% with no notable differences among the various tracers (4). The authors concluded that PSMA-directed PET agents were preferable to choline PET, particularly in patients with a serum PSA level of less than 1 ng/mL (4). However, to date, only single-center studies with a limited number of patients have compared ^18^F-fluorocholine with a radiolabeled PSMA ligand for PET/CT imaging of PCa recurrence localization, and large randomized controlled trials are lacking (5).

^18^F-PSMA-1007 was developed by Cardinale et al. (6) and Giesel et al. (7) in Heidelberg in 2016 as a PSMA-targeting ligand with low urinary excretion. The compound showed a high detection rate at low PSA values and high sensitivity and specificity in both biochemical recurrence and primary staging (8*,*^9^). In some patients, nonspecific bone uptake may be a confounding factor (10).

The ABX-CT-301 study (NCT04102553) aimed to compare ^18^F-PSMA-1007 PET/CT and ^18^F-fluorocholine PET/CT for the detection of PCa lesions in patients with biochemical recurrence. Secondary objectives were to compare the detection rates of the clinical investigators for both radiopharmaceuticals in a patient-based analysis; to assess the diagnostic performance of both radiotracers for PCa lesions in a region-based analysis; to assess the impact on diagnostic thinking, therapeutic decision making, and adequacy of therapy changes for both radiotracers; and to assess the safety profile of ^18^F-PSMA-1007.

## MATERIALS AND METHODS

### Population

This study was a prospective, open-label, randomized, 2-armed crossover study conducted in 6 study centers in France. Men who were at least 18 y old, who were diagnosed with PCa, and who had prior definitive therapy were considered for enrollment. Eligible patients presented with suspected PCa recurrence, defined as 3 consecutive PSA increases or a PSA rise of greater than or equal to 2.0 ng/mL above the nadir after radiotherapy (external-beam radiation therapy or brachytherapy) or cryotherapy or a PSA rise of greater than or equal to 0.2 ng/mL after prostatectomy. The main exclusion criteria were participation in another therapeutic clinical trial within 5 d of enrollment in the present study and a life expectancy of less than 6 mo.

The study protocol was approved by a national ethics committee certified by the French Ministry of Health (Institutional Review Board: IORG0009855). All patients gave written informed consent before randomization.

### Intervention

All men underwent both ^18^F-PSMA-1007 PET/CT and ^18^F-fluorocholine PET/CT using a standardized imaging protocol (supplemental materials, available at http://jnm.snmjournals.org). Patients were randomized using a computer-generated block-randomized sequence, stratified by center, to receive either ^18^F-PSMA-1007 PET/CT or ^18^F-fluorocholine PET/CT first. Patients underwent both PET/CT examinations within a minimum of 24 h and a maximum of 240 h; depending on the individual site preferences, either low-dose or diagnostic CT could be used, but the use of contrast agents was not permitted. At each PET/CT visit, vital signs were recorded before and after injection of the study drug and again at the end of the PET/CT examination. Samples for laboratory tests, including serum PSA levels, were obtained before the administration of each study drug. Patients were monitored for adverse events for 24 h after the second PET/CT examination (supplemental materials). Then we collected all data related to the treatments, additional diagnostic methods (including biopsy confirmation of detected foci, if feasible) and PSA values obtained in the 6 months during which the patients were followed.

### Image Reading and Standard of Truth

PET images were read on-site on the day of acquisition by investigators who were not masked regarding clinical data and were transferred to a core imaging laboratory where they were evaluated by 3 independent masked readers (supplemental materials). ^18^F-PSMA-1007 and ^18^F-fluorocholine images were read on separate days, at least 1 wk apart. The results of an “average reader” were determined statistically from the 3 independent reader results and not by a consensus read.

The composite standard of truth (recurrence, no recurrence, or undetermined) at the time of imaging was determined by an independent expert panel that considered all available clinical patient data from before inclusion to the end of the follow-up period, excluding all information from PET/CT (supplemental materials). The expert panel consisted of a urologist, a radiologist, and a nuclear physician; they reached their conclusions by consensus.

### Outcomes

The primary objective was to compare ^18^F-PSMA-1007 with ^18^F-fluorocholine with regard to the “correct detection rate” for recurrent PCa lesions on a patient basis, as determined by 3 independent readers and confirmed by an independent expert panel on the basis of a composite standard of truth (supplemental materials).

Secondary objectives were to compare the correct detection rates of the clinical investigators; to assess the correct detection rates of both radiotracers for PCa lesions in a region-based analysis; to report the impact on diagnostic thinking (impact of PET/CT result on posttest vs. pretest probability of a correct diagnosis), therapeutic decision making (impact on the comprehensive process by which physicians make decisions about the PCa response), and adequacy of management changes by the Investigators at 3 time points (before PET, immediately after both PET studies, and at the end of follow-up) and by the expert panel (only at the end of follow-up), using 3 dedicated forms (supplemental materials); to compare the masked intra- and interreader agreements; and to assess the safety profile of ^18^F-PSMA-1007.

### Statistical Analysis

Analysis was performed using SAS 9.4 or higher (SAS Institute), detailed in the supplemental materials. A *P* value of less than or equal to 0.05 was considered statistically significant. Descriptive statistics were calculated for quantitative variables; frequency counts by category were given for qualitative variables. Ninety-five percent CIs or interquartile ranges were given when appropriate. The intention-to-treat (ITT) population was the primary population for the analyses of efficacy endpoints and all baseline characteristics. The correct detection rate was determined for each reader individually. Generalized estimation equations were used to account for the correlations between readers’ assessments and to summarize the overall reader results (average reader). Patients for whom the expert panel could assess the true disease state on a patient level but for whom the affected region could not be identified by the expert panel were included as correct assessments on a patient basis. If, on a region basis, there was no region with a correct detection of recurrence compared with the standard of truth, then the patient was regarded as having a false-negative result. If at least in 1 region the reader and the expert panel detected a recurrence, independent of the other regions, then this result was classified as a true-positive result. Subgroup analyses were performed on the basis of the PSA level at baseline: less than 0.5 ng/mL, 0.5 ng/mL to less than 1.0 ng/mL, greater than or equal to 1.0 ng/mL to less than 2.0 ng/mL, and greater than or equal to 2.0 ng/mL. For the primary analysis on a patient level, 2 distinct analyses were performed by considering undetermined results as positive or negative for PCa recurrence. For subgroup and secondary analyses, undetermined results were considered negative for PCa recurrence. Intra- and interreader agreements were evaluated using pairwise and multiple Cohen κ-statistics. Each masked reader read 10% of the images twice (on separate occasions). Interreader agreement was assessed pairwise and across all 3 readers. The degree of agreement was defined as described by Landis and Koch (11).The sample size was selected to provide a power of at least 80% to detect a 10% difference in correct detection rates between the 2 products.

## RESULTS

### Population

**FIGURE 1.**
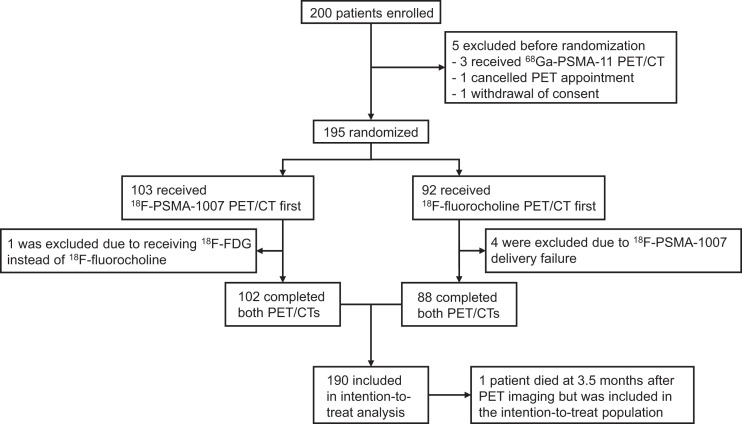
Trial chart.

From March 5, 2019, to October 8, 2020, 200 patients consented to this study; 195 were randomized, and 189 completed all follow-up assessments (Fig. 1). One patient ended study participation prematurely because he died 3.5 mo after PET imaging but was included in the ITT population. Most of the patients had previously undergone prostatectomy, and the median serum PSA level was 1.7 ng/mL. Study population characteristics are summarized in Table 1.

**TABLE 1.** Patient Characteristics (Intention-to-Treat Population)

Characteristic	All patients (*n* = 190)	^18^F-PSMA-1007 first (*n* = 102)	^18^F-fluorocholine first (*n* = 88)
Median age (y)*	69 (49–84)	68 (49–81)	70 (51–84)
Initial ISUP group grade at PCa diagnosis^†^			
1	29 (15.3)	12 (11.8)	17 (19.3)
2	64 (33.7)	32 (31.4)	32 (36.4)
3	55 (29.0)	33 (32.4)	22 (25)
4	14 (7.4)	8 (7.8)	6 (6.8)
5	21 (11.1)	13 (12.8)	8 (9.1)
Unknown	7 (3.7)	4 (3.9)	3 (3.4)
Prior prostatectomy^†^	154 (81)	80 (78)	74 (84)
With pelvic lymph node dissection (no. of patients)	93	51	42
Serum PSA levels, in ng/mL, before first PET examination^‡^			
Overall	1.7 (0.6–4.2)	2.0 (0.9–5.5)	1.3 (0.6–3.1)
In patients with prior prostatectomy	1.3 (0.5–3.2)	1.7 (0.6–3.5)	0.9 (0.4–2.5)
In patients without prior prostatectomy	4.5 (2.3–9.9)	6.3 (2.8–10.9)	3.0 (2.1–9.0)
Serum PSA doubling time, in mo, before first PET examination^‡^	6.3 (3–12.1)	6.4 (3.0–11.6)	5.9 (2.7–12.6)
PSA doubling time ≤ 6 mo (% of patients)	49	47	51
PSA doubling time ≤ 12 mo (% of patients)	74	76	70

* Values in parentheses are ranges.

† Reported as numbers of patients, with percentages in parentheses.

‡ Reported as medians, with interquartile ranges in parentheses.

ISUP = International Society of Urological Pathology.

The median follow-up period for the ITT population was 8.3 mo (range, 2.9–16.1 mo), as its duration was extended because of the COVID-19 pandemic.

### Primary Objective

Per-patient and per-region PET findings are detailed in the supplemental materials. At the patient level, the masked readers found evidence for PCa recurrence in 145–162 patients (76.3%–85.3%) with ^18^F-PSMA-1007 and 99–128 patients (52.1%–67.4%) with ^18^F-fluorocholine. Findings remained undetermined in 6–12 patients (3.2%–6.3%) with ^18^F-PSMA-1007 and 10–21 patients (5.3%–11.1%) with ^18^F-fluorocholine (supplemental materials). The expert panel confirmed PCa recurrence according to the standard of truth in 179 of 190 cases (94%).

The overall proportion of patients with correct detection rates for PCa lesions with ^18^F-PSMA-1007 was 0.82 (95% CI, 0.78–0.86) or 0.77 (95% CI, 0.72–0.82) when undetermined results were considered positive or negative for malignancy, respectively; these values were statistically superior to the value 0.65 (95% CI, 0.60–0.71) or 0.57 (95% CI, 0.51–0.62) obtained with ^18^F-fluorocholine when undetermined results were considered positive or negative for PCa, respectively (*P* < 0.0001) (Table 2). Thus, the primary endpoint was reached.

**TABLE 2.** Patient-Level Overall Proportion of Patients With Correct Rate of Detection of Recurrent PCa According to Standard of Truth and Positive Predictive Value (ITT Population) (*n* = 190)

Parameter	^18^F-PSMA-1007*	^18^F-fluorocholine*	*P*
Undetermined lesions considered positive for PCa recurrence in analysis			
Proportion	0.82 (0.78–0.86)	0.65 (0.60–0.71)	
Difference in proportion	0.16 (0.11–0.22)		<0.0001
Odds ratio	2.40 (1.79–3.21)		<0.0001
Positive predictive value	0.96 (0.93–0.99)	0.96 (0.93–0.99)	
Difference in positive predictive value	0.002 (0.031–0.035)		0.90
Odds ratio	0.95 (0.42–2.15)		0.90
Undetermined lesions considered negative for PCa recurrence in analysis			
Proportion	0.77 (0.72–0.82)	0.57 (0.51–0.62)	
Difference in proportion	0.21 (0.15–0.26)		<0.0001
Odds ratio	2.61 (1.97–3.46)		<0.0001
Positive predictive value	0.95 (0.92–0.99)	0.97 (0.95–1.00)	
Difference in positive predictive value	0.02 (0.01–0.05)		0.25
Odds ratio	0.58 (0.22–1.55)		0.27

* Values in parentheses are 95% CIs.

For both study drugs, the correct detection rate for PCa recurrence was higher for patients with higher PSA levels. For all examined PSA-level subgroups, the correct detection rate was statistically higher for ^18^F-PSMA-1007 (Table 3).

**TABLE 3.** Patient-Level Proportion of Patients With Correct Rate of Detection of PCa Lesions by PSA Level at Baseline (ITT Population) (*n* = 190)

Parameter	^18^F-PSMA-1007*	^18^F-fluorocholine*	*P*
PSA < 0.5 ng/mL			
No. of patients with recurrence detected by SOT = 43			
Proportion	0.57 (0.45–0.68)	0.39 (0.28–0.50)	
Odds radio	2.10 (1.13–3.89)		0.002
0.5 ng/mL ≤ PSA < 1.0 ng/mL			
No. of patients with recurrence detected by SOT = 25			
Proportion	0.83 (0.72–0.93)	0.43 (0.28–0.58)	
Odds radio	6.88 (3.35–14.13)		<0.0001
1.0 ng/mL ≤ PSA < 2.0 ng/mL			
No. of patients with recurrence detected by SOT = 33			
Proportion	0.81 (0.72–0.89)	0.50 (0.37–0.62)	
Odds radio	4.31 (2.26–8.24)		<0.0001
PSA ≥ 2.0 ng/mL			
No. of patients with recurrence detected by SOT = 78			
Proportion	0.85 (0.79–0.91)	0.74 (0.66–0.82)	
Odds radio	2.01 (1.27–3.19)		0.003

* Values in parentheses are 95% CIs.

SOT = standard of truth.

Masked intra- and interreader agreements for the detection of metastases (patient level) ranged from 0.24 to 0.73 and from 0.30 to 0.36 for ^18^F-PSMA-1007 and from 0.48 to 0.72 and 0.34 to 0.40 for ^18^F-fluorocholine, respectively (supplemental materials).

### Secondary Objectives

#### Comparison of Patient-Based Correct Detection Rates According to Investigator Findings

On the basis of the clinical investigators’ overall findings, the correct detection rates were 0.80 (95% CI, 0.74–0.86) for ^18^F-PSMA-1007 and 0.50 (95% CI, 0.42–0.57) for ^18^F-fluorocholine (*P* < 0.0001).

#### Comparison of Region-Based Correct Detection Rates According to Masked Readers’ Findings

In the 72 patients for whom 1 or more regions could be assessed by the expert panel, there were 78 regions with confirmed PCa lesions. The most common sites for PCa lesions were the pelvis (59 patients) and the spine (6 patients) (supplemental materials). In the patients considered to have a positive PET result, more suggestive lesions were detected with ^18^F-PSMA-1007 than with ^18^F-fluorocholine, especially in patients with 3 or more lesions.

Overall composite region-level sensitivities were 0.77 (95% CI, 0.69–0.84) for ^18^F-PSMA-1007 PET and 0.57 (95% CI, 0.48–0.67) for ^18^F-fluorocholine PET (*P* < 0.0001).

#### Impact on Diagnostic Thinking, Therapeutic Decision Making, and Adequacy of Therapy Changes

The investigator assessments of the changes in diagnostic thinking after both PET/CT examinations and at the end of follow-up are summarized in Tables 4 and 5 and the supplemental materials. Treatment plans before and after PET/CT examinations were available in 187 patients. Treatment plans were changed in 100 patients; 89 of the changes were major (supplemental materials). Changes in diagnostic thinking due to PET/CT were reported in 149 patients. Diagnostic thinking was unchanged in 41 patients (including 3 with no reported answer in the questionnaire). In the 149 patients for whom there were changes in diagnostic thinking, ^18^F-PSMA-1007 contributed more in 93 patients (62%), both tracers contributed equally in 49 patients (33%), and ^18^F-fluorocholine contributed more in 4 patients (3%).

**TABLE 4.** Change in Diagnostic Thinking After Both PET/CT Scans (ITT Population) (*n* = 190)*

Change in diagnostic thinking	^18^F-fluorocholine examination contributed more	^18^F-PSMA-1007 examination contributed more	Both PET examinations contributed equally	Missing
Yes				
PET identified site of recurrence that was not known before	3 (1.6)	80 (42.1)	29 (15.3)	3 (1.6)
PET confirmed site of recurrence that was suspected before	1 (0.5)	6 (3.2)	3 (1.6)	
Other		4 (2.1)	15 (7.9)	
Missing		3 (1.6)	2 (1.1)	
				
No		38 (20)		
Missing				3 (1.6)

* Data are reported as numbers of patients, with percentages in parentheses.

**TABLE 5.** Change in Diagnostic Thinking After Both PET Scans and Influence at End of Follow-up (ITT Population) (*n* = 190)*

	Influence was:			
Category	To benefit of patient	Not to benefit of patient	Neither to benefit nor disadvantage of patient	Missing
^18^F-fluorocholine examination contributed more				
More accurate diagnosis	6 (3.2)	0	0	0
Diagnostic thinking was misled by PET	0	0	0	0
PET had no influence	0	1 (0.5)	1 (0.5)	0
Missing	0	0	0	0
^18^F-PSMA-1007 examination contributed more				
More accurate diagnosis	88 (46.3)	2 (1.1)	10 (5.3)	2 (1.1)
Diagnostic thinking was misled by PET	1 (0.5)	1 (0.5)	2 (1.1)	0
PET had no influence	0	0	1 (0.5)	0
Missing	0	0	0	0
Both PET examinations contributed equally				
More accurate diagnosis	27 (14.2)	0	13 (6.8)	0
Diagnostic thinking was misled by PET	0	5 (2.6)	1 (0.5)	0
PET had no influence	5 (2.6)	2 (1.1)	16 (8.4)	0
Missing	0	0	1 (0.5)	0
Missing				
More accurate diagnosis	1 (0.5)	0	0	0
Diagnostic thinking was misled by PET	0	0	0	0
PET had no influence	0	0	0	0
Missing	0	0	0	4 (2.1)

* Data are reported as numbers of patients, with percentages in parentheses.

In 122 patients, PET/CT led to a more accurate diagnosis that benefited them. In 11 patients, PET/CT was not to the benefit of the patient, and in 45 patients, PET/CT did not exert a positive or negative influence. In the 122 patients with a more accurate diagnosis after PET/CT that benefited them, ^18^F-PSMA-1007 contributed more in 88 patients, ^18^F-fluorocholine contributed more in only 6 patients, and both contributed equally in 27 patients.

#### Safety Profile of ^18^F-PSMA-1007

There were no serious adverse events. No patient discontinued study participation because of an adverse event. Four patients had 4 events (toothache, diarrhea, chest discomfort, and arterial hypertension) after the administration of ^18^F-PSMA-1007, and 1 patient had 1 event (shoulder pain) after the administration of ^18^F-fluorocholine. None of the adverse events was considered to be attributable to the study drug.

## DISCUSSION

To our knowledge, the present study is the first multicenter, crossover randomized study to compare ^18^F-PSMA-1007 and ^18^F-fluorocholine for the localization of biochemical recurrence of PCa. The correct detection rate was significantly higher with ^18^F-PSMA-1007 PET/CT than with ^18^F-fluorocholine PET/CT. Results were similar for the population as a whole and for the 72 patients for whom individual lesions could be verified, either by biopsy or response to local treatment. The difference was especially pronounced in patients with lower serum PSA levels, allowing earlier, targeted salvage treatment. Our results for ^18^F-PSMA-1007 are in agreement with those reported in the literature for ^68^Ga-PSMA-11 (4*,*^12^–^18^). In a metaanalysis, Treglia et al. (19) also found similar results when comparing a PSMA tracer (^68^Ga-PSMA-11 or ^64^Cu-PSMA-617) and radiocholine. Because the results obtained with the various PSMA ligands are generally similar, it is generally accepted that the PSMA ligands are interchangeable for this indication. PSMA ligands radiolabeled with ^18^F have wider accessibility than those radiolabeled with ^68^Ga.

The investigator assessments after PET/CT and at the end of follow-up demonstrated the superiority of ^18^F-PSMA-1007 over ^18^F-fluorocholine in identifying sites of recurrence. The impact of ^18^F-PSMA-1007 was to benefit the patient in most cases. These results are likely linked to the higher correct detection rate for ^18^F-PSMA-1007. In previous studies with ^18^F-fluorocholine (13*,*^20^) and ^68^Ga-PSMA-11 (5*,*^21^), rates of impact on patient management of 39%–58% were reported. Our study is consistent with published data on ^18^F-fluorocholine and demonstrates a higher impact of ^18^F-PSMA-1007 on patient management.

The strengths of our study are its prospective, multicenter, randomized crossover design. Limitations include the lack of histopathology for most lesions. Because obtaining histopathology is often ethically questionable or medically impractical, our standard of truth was a composite based on biopsy, response to local therapy, imaging, and changes in serum PSA levels during 6 mo of follow-up, as established by an independent panel of experts. The use of the independent panel removed potential bias in determining “truth” while modeling what is done in “real-life” practice.

**FIGURE 2.**
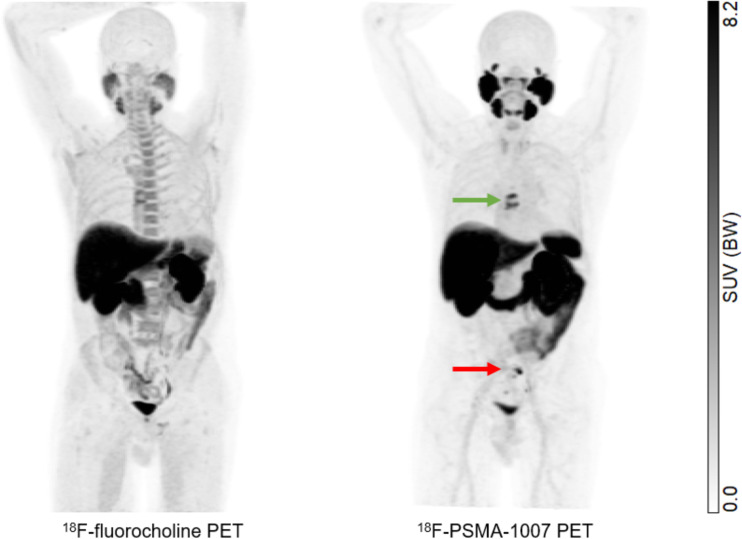
62-y-old patient with history of PCa (International Society of Urological Pathology grade 3; PSA level of 5.7 ng/mL), initially treated with prostatectomy (pT3N0R0), prostate bed radiation therapy, and 6 mo of androgen deprivation therapy (ADT), presenting with PSA recurrence (0.72 ng/mL). ^18^F-PSMA-1007 PET/CT detected pelvic lymph nodes (red arrow) and bone metastases (green arrow) that were not detected by ^18^F-fluorocholine PET/CT. Therapeutic management changed from targeted radiation therapy before PET to ADT after PET, leading to drop in PSA level to 0.1 ng/mL at 6 mo. BW = body weight.

In this work, we found that ^18^F-PSMA-1007 had an impact on diagnostic thinking and therapeutic decision making and that therapy changes were more beneficial when based on ^18^F-PSMA-1007 PET/CT findings than when based on ^18^F-fluorocholine PET/CT findings (Fig. 2). However, we did not make a statistical comparison of these data because the study was not powered for this purpose.

## CONCLUSION

This prospective, multicenter, open-label, crossover randomized study demonstrated that ^18^F-PSMA-1007 PET/CT localizes PCa recurrence in significantly more patients than ^18^F-fluorocholine PET/CT, especially in patients with low PSA serum levels. ^18^F-PSMA-1007 PET/CT also had a higher impact on diagnostic thinking, therapeutic decision making, and therapy changes than ^18^F-fluorocholine PET/CT.

## DISCLOSURE

This study was funded and supported by ABX Advanced Biochemical Compounds, Radeberg, Germany. Ines Rudolph and Alexander Hoepping work for ABX Advanced Biochemical Compounds. Charles Merlin has received personal fees and nonfinancial support from Curium Pharma and Novartis. Mathieu Gauthé has received personal fees and nonfinancial support from Astellas, Curium Pharma, and Novartis. The manuscript was edited for English language by Dr. Jay R Wiggins, PhD, and Dr. Kelsey L. Pomykala, MD, supported by ABX Advanced Biochemical Compounds. The ABX-CT-301 ClinicalTrials.gov number is NCT04102553. No other potential conflict of interest relevant to this article was reported.

## Notes

KEY POINTS **QUESTION:** Is ^18^F-PSMA-1007 superior to ^18^F-fluorocholine PET/CT for the localization of biochemical recurrence in PCa patients? **PERTINENT FINDINGS:** In this prospective, open-label, randomized, crossover multicenter study that included 190 patients with PCa biochemical recurrence, we demonstrated that ^18^F-PSMA-1007 PET/CT localizes significantly more PCa lesions than ^18^F-fluorocholine PET/CT, especially when serum PSA levels are low. **IMPLICATIONS FOR PATIENT CARE:** More accurate staging of recurrent PCa might lead to more beneficial decision making and patient management; a theranostic use of PSMA radioligands should be considered in recurrent PCa.
